# PERSON-CENTERED ONLINE DEMENTIA TRAINING

**DOI:** 10.1093/geroni/igae098.1100

**Published:** 2024-12-31

**Authors:** Diana Sturdevant, Kimethria Jackson, Kimetha Broussard

**Affiliations:** University of Oklahoma Health Sciences Center, Oklahoma City, Oklahoma, United States; University of Oklahoma Health Sciences Center, Oklahoma City, Oklahoma, United States; University of Oklahoma Health Sciences Center, Oklahoma City, Oklahoma, United States

## Abstract

Nursing education that focuses on care of people living with dementia (PLWD), and training in person-centered dementia care is limited. Person-centered dementia care puts PLWD at the center of the care process. It requires deep knowledge of the individual, as well as empathy and understanding from the nurse. Person-centered dementia care training improves care by correcting misconceptions about dementia and increases confidence in managing behavioral-psychological symptoms of dementia. In this session we will report outcomes from an on-line training focused on improving nursing care of people living with dementia (PLWD). The training consisted of 4 modules that included multimedia, discussion boards, and interactive exercises to stimulate student engagement and interaction. Undergraduate nursing students were recruited via email announcements of the volunteer opportunity. A total of (n=31) participants were recruited from 4 baccalaureate nursing programs including Traditional Junior, Traditional Senior, Degree Completion LPN to BSN, and Accelerated BSN. Pre/post assessments included the Dementia Attitudes Scale, the ADRC Staff Dementia Knowledge Assessment Tool, and the Confidence in Dementia Scale. A 5pt-likert scale was used to evaluate course structure and content, and open-ended questions provided prompts for additional participant comments. Common themes from evaluations included: need for additional content, more interactive activities, and inclusion as a required course for all nursing students.

